# Preparation and Characterization of Quartz-Reinforced Hybrid Composites Based on Unsaturated Polyester Resin from Post-Consumer PET Recyclate

**DOI:** 10.3390/ma17051116

**Published:** 2024-02-28

**Authors:** Przemysław Pączkowski, Karolina Głogowska

**Affiliations:** 1 Department of Polymer Chemistry, Institute of Chemical Sciences, Faculty of Chemistry, Maria Curie-Skłodowska University, Gliniana 33, 20-614 Lublin, Poland; 2 Department of Technology and Polymer Processing, Faculty of Mechanical Engineering, Lublin University of Technology, Nadbystrzycka 36, 20-618 Lublin, Poland; k.glogowska@pollub.pl

**Keywords:** hybrid composite, unsaturated polyester resin, quartz flour, mechanical properties, thermal properties, surface properties

## Abstract

The paper presents the results of research on hybrid composites made of unsaturated polyester resin based on post-consumer recycled poly(ethylene terephthalate). The polymeric materials were reinforced with quartz flour, which is a common inorganic mineral filler. An environmentally friendly cobalt polymer solution was used to cure the polyester matrix. The results showed the quantitative influence of the quartz filler on the thermal, mechanical and morphological properties of the quartz–polyester composites. A change in the surface wettability and the polarity of the polymeric materials was also noticed, with some deterioration of their gloss.

## 1. Introduction

Due to their high-performance properties and low cost, unsaturated polyester resins (UPRs) are one of the most frequently used thermosetting resins, among others, in the marine and automotive industries. UPRs can be prepared according to the condensation reaction of aliphatic diols (glycols) with unsaturated acid and saturated diacids. By diluting the synthesized polyester in a cross-linking monomer, most often styrene, an unsaturated resin is obtained [1,2].

Unsaturated polyester composites, due to their unique engineering properties, are increasingly replacing traditional materials such as wood, glass and metal in construction, furniture, machine and vehicle components, as well as in other consumer products [3].

Due to its cost-effective product for producing polymer composites, recently, unsaturated polyester resin from recycled poly(ethylene terephthalate) (rPET) has attracted the attention of many researchers [4,5,6,7].

Polyester resins come in two forms, orthophthalic and isophthalic, depending on the compound used for synthesis [8]. Produced from phthalic acid, orthophthalic polyester resin is an inexpensive general-purpose resin with a styrene content ranging from 35 to 45%. This resin type is primarily utilized in applications that do not have to be resistant to corrosion and elevated temperatures or have high mechanical properties. In contrast, the other resin type, which is produced from isophthalic acid, is relatively more expensive and has a styrene content ranging from 42 to 50%. Not only is isophthalic polyester resin resistant to corrosion but it also exhibits high thermal and mechanical properties.

Quartz is often used as a filler in polymer matrices such as polyethylene [9,10], polypropylene [11,12], poly(lactic acid) [13], poly(ethylene glycol) [14], poly(methyl methacrylate) [15], polyester resin [16], epoxy resin [17,18] and vinyl ester resin [19].

Quartz-reinforced polyester composites have been successfully produced according to the compression molding of quartz after its impregnation with an unsaturated polyester resin. When the amount of quartz in a composites changes, there is a significant difference in its physical, mechanical and thermal properties. Unsaturated polyester has higher reactivity and, with its generally lower cost than epoxy resin, is one of the most commonly used resins for the production of lightweight polymer concrete [8,20,21].

The use of polyester resins reinforced with a mineral filler such as quartz flour involves the use of resulting materials such as artificial stone or marble, monolithic flooring, polymer-modified mortar and concrete and mine charges [4,8,20,21,22,23].

Polymer-based binders cause materials to have better tensile properties and a better cracking strength, chemical resistance and durability compared to classic Portland-based cement [21].

In general, the use of recycled polymers and various types of additives, cheap materials found in nature, makes synthetic products or plastics not only more environmentally friendly but also more desirable from an economic point of view.

The integration of these natural fillers with recycled polymers not only fits into the global drive to increase the use of renewable materials and secondary resources but can also contribute to improving some of the mechanical and thermal properties of the finished products. Moreover, this innovative approach represents a step forward in minimizing the negative impact of industry on the environment, promoting a circular economy.

The results of this research are expected to not only contribute to the development of new, greener composite materials but also open up new opportunities for the recycling industry, construction and many other sectors looking for durable and sustainable material solutions.

The work attempts to assess the thermal, mechanical and morphological properties of hybrid composites made of unsaturated polyester resin based on post-consumer PET recyclate. The influence of quartz flour’s addition to polyester materials on their surface gloss, wettability and polarity was studied and discussed.

## 2. Materials and Methods

### 2.1. Materials

In the study, we used Estromal 14.PB-06, which is a commercially available unsaturated orthophthalic polyester resin obtained from post-consumer recycled poly(ethylene terephthalate) from drink bottles. The resin was supplied by LERG S.A. (Pustków, Poland). This blue–greenish syrup-like liquid has an acidic number of 22.3 mg KOH g^−1^, a viscosity of 230 mPas at 23 °C, a non-volatile content of 57.8% and a reactivity factor of 2.0.

The curing process was conducted using Luperox DHD-9 (2-butanone peroxide solution from Sigma-Aldrich (St. Louis, MO, USA)) and 4% polymeric cobalt solution. The polymeric cobalt was prepared at the Department of Polymer Chemistry of Maria Curie-Skłodowska University using the procedure described in a patent [24].

In this study, we used a natural mineral filler in the form of quartz flour (QF), marketed under the trade name MK.075/001. The filler is produced by SKSM Sobótka—Strzeblowskie Kopalnie Surowców Mineralnych Sp. z o.o. (Sobótka, Poland). This quartz filler is manufactured from natural quartz sand by means of iron-contamination-free milling and air classification. According to the manufacturer’s data and the chemical parameters of the product (wt.%), the main component of the powder is SiO_2_ (min. 99.0%). The filler also contains trace amounts of Al_2_O_3_ (max. 0.50%), Fe_2_O_3_ (max. 0.05%) and TiO_2_ (max. 0.05%). 

Quartz flour is characterized by a whitish–light gray color. The standard density and the bulk density of this mineral filler are 2650 kg·m^−3^ and 756 kg·m^−3^, respectively.

Using a laser diffraction particle size analyzer, the following d10, d50 and d90 results were obtained for the quartz flour: 4.58 μm, 27.64 μm and 83.03 μm, respectively. These are statistical parameters that were read directly from the cumulative particle size distribution. They indicate the size below which 10%, 50% or 90% of all particles are found. 

Quartz flour particles have an irregular shape, and their permissible residue on a 71 µm control sieve is about 10 wt.%.

### 2.2. Preparation of the Polymer Composites and Their Samples

The polymer composites were prepared by mixing the unsaturated polyester resin (UPR) with different amounts of quartz flour (5, 10, 20 and 40 wt.%) relative to the UPR weight. Based on the resin content, 1.1 wt.% of the initiator (Luperox) and 0.25 wt.%. of the accelerator (polymeric cobalt solution) were added.

The homogeneously prepared mixture was degassed with a vacuum set from VacuumChambers.eu (Białystok, Poland). The contents were then poured into glass molds using a U-shaped Teflon spacer. The prepared composites were left to cure at room temperature for 24 h and then post-cured in an oven at 80 °C for 10 h.

Cuboid-shaped composite sections with dimensions of 80 mm × 10 mm × 4 mm (thickness) were cut from the molding using a CNC milling machine, MFG 8037P from Ergwind (Gdańsk, Poland).

### 2.3. Methods

#### 2.3.1. Morphological Characterization

The Mastersizer 2000 from Malvern Panalytical (Malvern, UK) was used to determine the size of the quartz flour particles. A laser particle size analyzer was operated in a range from 0.02 to 2000 µm, which determines not only the size of the filler but also its size distribution. An adapter for dynamic measurements in liquid dispersions was applied. The quartz flour was pre-dispersed in distilled water and sonicated for 10 min. Then, using water as the dispersing phase, measurements of the wet filler were taken.

To analyze the shape of the dry quartz powder particles, the Morphologi G3 microscopic device from Malvern Panalytical (Malvern, UK) was used.

To determine the dry quartz flour fraction, the test sieve shaker EML 200 Premium from HAVER & BOECKER (Oelde, Germany) was applied.

#### 2.3.2. Thermal Characterization

The thermal properties of the UPR-based composite samples were assessed using thermogravimetry. The TG/DTG data were acquired using the STA 449 F1 Jupiter thermal analyzer from Netzsch (Selb, Germany) at a temperature ranging from 30 to 1000 °C and a heating rate of 10 °C min^−1^ in an oxidative atmosphere of synthetic air. The test procedure complied with the requirements specified in the EN ISO 11358-1:2014 standard [25].

The Vicat softening temperature (VST) was determined using a CEAST HV3 tester from Instron (Turin, Italy) according to the EN ISO 306:2013 standard [26]. The B120 measurement method was applied, with a force of 50 N and a heating rate of 120 °C h^−1^.

The heat deflection temperature (HDT) test was performed using the CEAST HV3 Instron apparatus (Turin, Italy) in accordance with the EN ISO 75-2:2013 standard [27]. The C-measurement method (flexural stress of 8 MPa) with a heating rate of 120 °C h^−1^ was used. A deflection value of 0.36 mm was also assumed.

#### 2.3.3. Mechanical Characterization

The impact of the quartz flour on the UPR composite hardness was assessed using the 7206/H04 Shore durometer from Zwick (Ulm, Germany). The assessment was made in compliance with EN ISO 868:2003, at a standard temperature of 23 °C ± 2 °C [28]. After 15 s of resin molding, five individual measurements were arithmetically averaged.

The mechanical properties of the UPR/quartz flour composite samples were determined at room temperature using the Zwick/Roell Z010 universal testing machine from Zwick GmbH Co (Ulm, Germany), with a load capacity of 10 kN. The test samples had dimensions of 80 mm × 10 mm × 4 mm. Their properties were assessed via a three-point bending flexural test conducted at a speed of 5 mm min^−1^ and with a 64 mm span between the supports, in accordance with the EN ISO 178 standard [29]. The following were measured in the test: flexural strain at break ($\varepsilon_{f}$), flexural modulus ($E_{f}$) and flexural strength ($\sigma_{f}$). 

Composite samples with dimensions of 80 × 10 × 4 mm were used for the Charpy unnotched impact test. The measurement was carried out in accordance with the ISO 179-2:2020 standard [30] using a 639F type impact hammer from Cometech Testing Machines (Taizhong, Taiwan), where the pendulum used had a maximum energy of 5 J.

#### 2.3.4. Surface Characterization

Gloss measurement was performed using a Zehntner ZGM 1110 triple-angle gloss meter from Zehntner GmbH Testing Instruments (Sissach, Switzerland). This device allows the simultaneous display of values for 20°, 60° and 85° geometries, suitable for surfaces from high-gloss to matte. These determinations were made in accordance with the ASTM D2457 standard [31], where the gloss values for the standard of a highly polished black glass were 86.8 GU (20°), 93.4 GU (60°) and 99.7 GU (85°).

Evaluation of the surface free energy (SFE) was determined according to direct contact angle measurements using the goniometer OCA 15EC from DataPhysics Instruments GmbH (Filderstadt, Germany) and the drop shape analyzer DSA100S from KRÜSS GmbH (Hamburg, Germany). Distilled water and diiodomethane (CH_2_I_2_) were used as the standard test liquids, with known values for the polar and dispersive components of the surface tension (Table 1). Measurement of the contact angles for the standard test liquids makes it possible to determine the total solid surface free energy and its dispersive and polar components. In this study, the measurements were made using a 2 μL droplet volume. The contact angles were captured using a CCD camera and measured 5 s after droplet formation. After that, the average values of ten droplets of the same sample on different areas were used for calculating the surface free energy. The contact angles were measured at a temperature of 21 ± 1 °C and an air humidity of 30 ± 1%.

The Owens–Wendt calculation method was applied to the contact angle data of the test liquids in order to evaluate the parameters of the SFE (Equation (1)) [32,33,34]:

(1) $$\gamma_{S}=\gamma_{S}^{d}+\gamma_{S}^{p},$$

where:

$\gamma_{S}$—total surface free energy,
$\gamma_{S}^{d}$—dispersive component of the surface free energy of the tested materials,
$\gamma_{S}^{p}$—polar component of the surface free energy of the tested materials.

From Equations (2) and (3), the dispersive and polar components of the surface free energy at the interface can be calculated:

(2) $${\left(\gamma_{S}^{d}\right)}^{0.5}=\frac{\gamma_{d}\left(cos\theta_{d}+1\right)-\sqrt{\frac{\gamma_{d}^{p}}{\gamma_{w}^{p}}}\gamma_{w}(cos\theta_{w}+1)\;}{2\left(\sqrt{\gamma_{d}^{d}}-\sqrt{\gamma_{d}^{p}\frac{\gamma_{w}^{d}}{\gamma_{w}^{p}}}\right)},$$

(3) $${\left(\gamma_{S}^{p}\right)}^{0.5}=\frac{\gamma_{w}\left(cos\theta_{w}+1\right)-2\sqrt{\gamma_{S}^{d}\gamma_{w}^{d}}}{2\sqrt{\gamma_{w}^{p}}},$$

where:

$\gamma_{d}$—surface free energy of diiodomethane,
$\gamma_{d}^{d}$—dispersive component of the diiodomethane surface free energy,
$\gamma_{d}^{p}$—polar component of the diiodomethane surface free energy,
$\gamma_{w}$—surface free energy of distilled water,
$\gamma_{w}^{d}$—dispersive component of the distilled water surface free energy,
$\gamma_{w}^{p}$—polar component of the distilled water surface free energy,
$\theta_{d}$—the value of the contact angle measured for diiodomethane,
$\theta_{w}$—the value of the contact angle measured for distilled water.

## 3. Results and Discussion

The polymer materials obtained by mixing quartz flour with unsaturated polyester resin were examined in terms of their physicochemical, thermal and mechanical properties for future potential use. The influence of the filler’s incorporation on the surface, i.e., the gloss and wettability of the tested products, was also determined.

Quartz is used as a filler not only to improve strength and regulate viscosity but it also produces smooth surfaces and is extremely weather-resistant compared to sand (which is composed of various minerals, such as quartz, feldspar, mica and other silicate minerals) [35]. This stems from the fact that quartz contains a particular crystalline form of SiO_2_. In turn, commercial silica contains a considerable amount of silanol groups (on the surface) that form hydrogen bonds, primarily with adsorbed water [36]. As a result of the formation of hydrogen bonds between the silanol groups on the quartz surface and the soft segments of ester carbonyl groups, the addition of quartz leads to enhancing the thermal, rheological, mechanical and adhesive properties of the polyester [35,36,37]. Significantly, the occurrence of a silane coupling agent in high-molecular-weight composites is also characterized by the fracture toughness, flexural and compressive loading modes of the composites [38]. 

**Figure 1:**
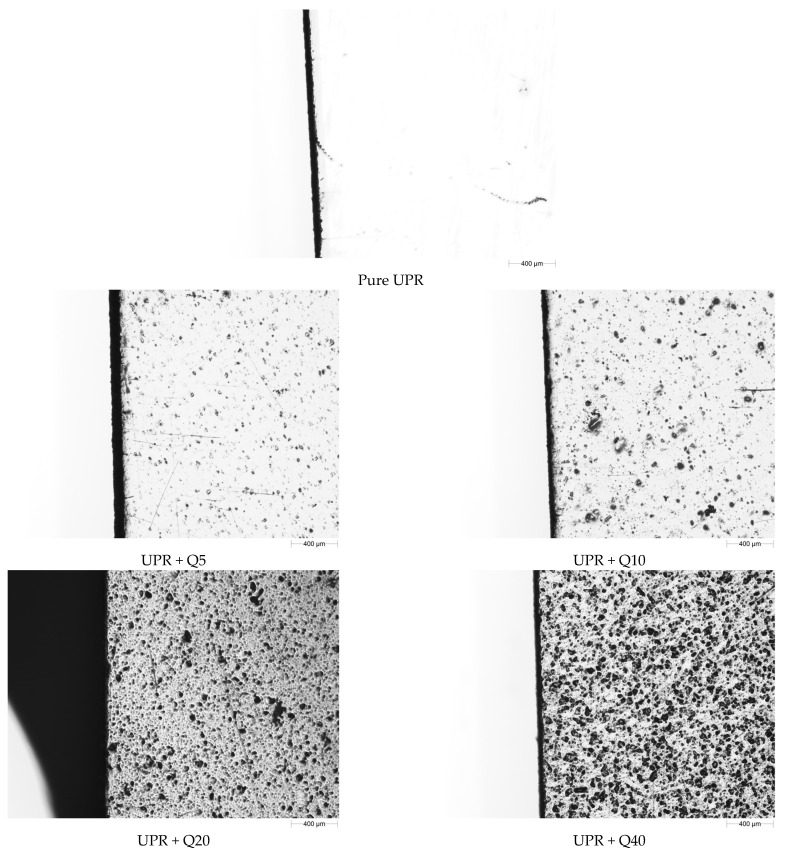
Micrographs of the surface of quartz–resin hybrid composites at magnification 2.5×.

Using the top light source, the micrograph taken up for the pure UPR specimen reveals a smooth surface and sometimes only river lines (Figure 1). As the amount of quartz flour in the hybrid composites increases, the surface of the material becomes rougher with more pits and holes, quite similar to the semi-porous surface form, especially for UPR + Q40.

Table 2 presents the results of the thermal property studies of the pure UPR and its hybrid composites with quartz flour. 

**Figure 2:**
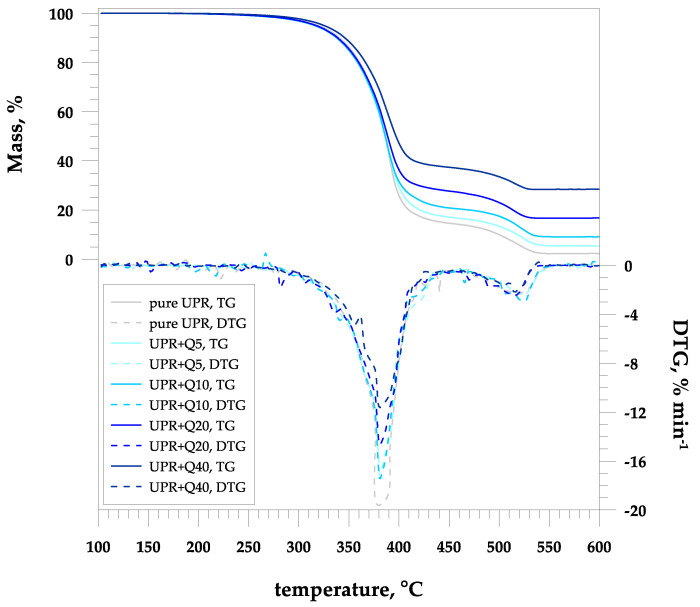
Thermal decomposition of the pure UPR and its hybrid composites with quartz flour.

From the TG–DTG curves, it can be seen that the pure polyester starts to decompose in air at a temperature of approximately 250 °C and has two degradation steps, with the decomposition maxima at 382 and 519 °C (Table 2, Figure 2). The decomposition of pure polyester occurs mainly as a result of scission the ester bonds in its polymer network, which was discussed in more detail in a previous work [39]. The first signals indicating the decomposition of the resin matrix come from ester bonds, specifically their aliphatic chains and aromatic rings (phthalic derivatives). Degradation at higher temperatures caused by the presence of an oxidizing atmosphere is associated with the combustion process, during which the chain of polyester and polystyrene fragments is further broken.

Quartz is a very thermally resistant material, and only its structure changes upon heating into various SiO_2_ polymorphs, i.e., a phase transformation from α-quartz to β-quartz [40,41].

In the case of the UPR-based hybrid composites, two stages of degradation were similarly observed to the case of pure polyester. As expected, increasing the quartz flour content from 5 to 40% results in an increase in the residual mass from 4.87 to 28.58%. In the case of the hybrid composite materials, due to the thermal stability of the quartz powder up to 1000 °C, only accidental chain-breaking, cyclization or scission of the ester bond of the polyester matrix take place as a result of thermo-oxidative degradation.

At the same time, the filler loading in the composite affects the characteristic temperature. The temperature values at 5%, 10% and 50% mass loss of the pure polyester were 317, 338 and 386 °C, respectively, and gradually raised to 325, 346 and 397 °C with the increase in the amount of the quartz flour. The values of the maximum of the decomposition temperature for the samples with the increasing filler incorporation stayed stable in the first area (382–384 °C), and in the second one, they slightly decreased from 519 to 514 °C. The reinforcement in the composite materials caused some delay at the start of the scissioning of polyester chains. However, once this process began, the decomposition of the composite materials was faster and completed at slightly lower temperatures. The decomposition temperature decreases in the presence of an inorganic filler, which suggests that the filler affects the thermal degradation mechanism of the polymer and inorganic particles reduce the thermal stability of the polymer composites [42]. It is believed that the main cause of thermal instability is probably the effect of indirect, improved and more effective heat transfer to the polymer matrix through the dispersed inorganic phase.

**Figure 3:**
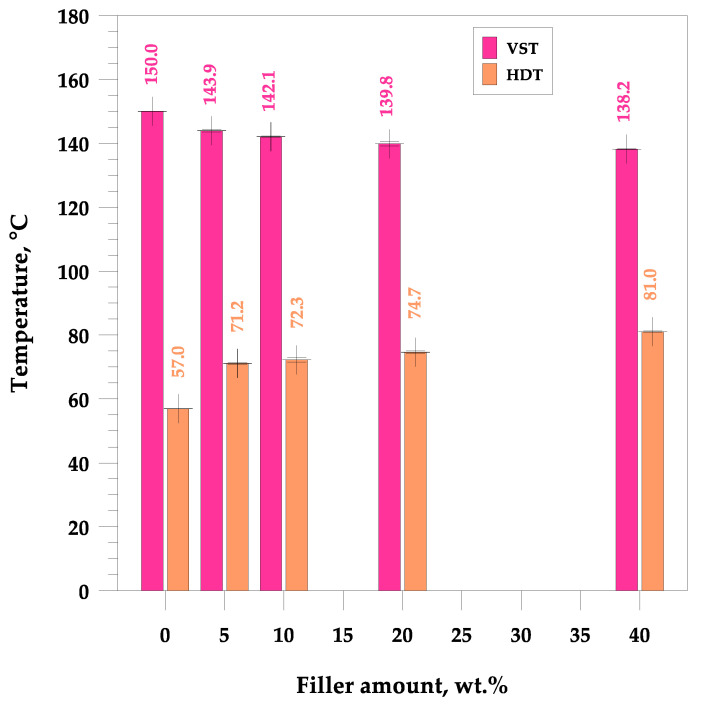
Effect of the filler’s incorporation on the characteristic temperatures. Vicat softening (VST) and heat deflection (HDT).

In the case of pure UPR, the Vicat tester is unable to detect temperatures above 150 °C, which is the maximum temperature to which silicone oil can be heated in the Instron CEAST HV3 oil bath tester, where the needle penetrated to a depth of 0.72 mm. As the quartz filler content increases, the VST temperature steadily decreases to 138.2 °C for the hybrid composite with the highest amount of additive (Figure 3).

The opposite situation occurred in the case of the heat deflection temperature, the value of which constantly increased from 57.0 °C for the pure polyester to 81.0 °C for the hybrid quartz-reinforced composite based on polyester resin (UPR + Q40).

Table 3 shows the hardness of the pure polyester and its quartz-reinforced composites. The results of the other mechanical studies of the specimens determining the impact strength and flexural properties are also presented.

The adhesion between the matrix and the reinforcing particles plays a key role in the load transfer, which means that poor adhesion results in failure of the load transfer. The reinforced samples have a rough fracture surface and relatively good adhesion to the polyester matrix. Thus, the deterioration of the polyester network and the presence of the filler aggregates caused the significant changes in the mechanical properties of the hybrid composites [43].

A Charpy hammer was used to test the impact strength of the quartz-reinforced composites. The inorganic mineral filler reduced the impact strength tendency in the polyester composites. The value changed slightly by 2% for a small amount of quartz flour, then drastically by 26.8%, 38.9% and 48.7% for 10, 20 and 40 wt.%, respectively. Polyester composites have relatively brittle properties (hard and rigid but, at the same time, has little tensile strength) with a low impact strength compared to a reference sample consisting of only pure polyester.

The hardness value ranges from 72.1 °ShD for pure polyester to 75.3 °ShD for the hybrid composite with the maximum amount of filler (Table 3). Because the stiffness of the quartz filler is greater than that of the polyester matrix, quartz–resin composites are harder than pure polyester.

The flexural properties of the pure UPR, such as modulus, strength and strain, at break were 3.66 GPa, 76.58 MPa and 2.12%, respectively. 

With an increasing amount of the quartz filler, the flexural modulus value continuously increases up to 5.26 GPa for UPR + Q40. The situation regarding the flexural strength and strain at break was completely different. First, the values increased for 5 wt.% of the filler to 93.20 MPa and 2.51%, respectively, and then decreased with the increase in the quartz content to 71.99 MPa and 1.41%, respectively, for UPR + Q40. As a result of the formation of hydrogen bonds between the silanol groups present on the quartz surface and the soft segments of the ester carbonyl groups of the resin, a small addition of quartz leads to the improved mechanical properties of the polyester. However, as the reinforcing filler ratio increases, agglomeration may occur, and this phenomenon may be the main reason for the reduction in the mechanical strength of the composites.

The direct relationship between the mechanical properties and the quartz content can be attributed to the degree of adhesion of the polymer to the filler. In the case of high content, the mechanical properties deteriorated due to the effect on the cross-linking density and the increase in the distance between the polymer chains and the quartz particles, which affect the contact between them. It can also be concluded that with a high filler content, the counteracting particles act as stress concentrators [44].

**Figure 4:**
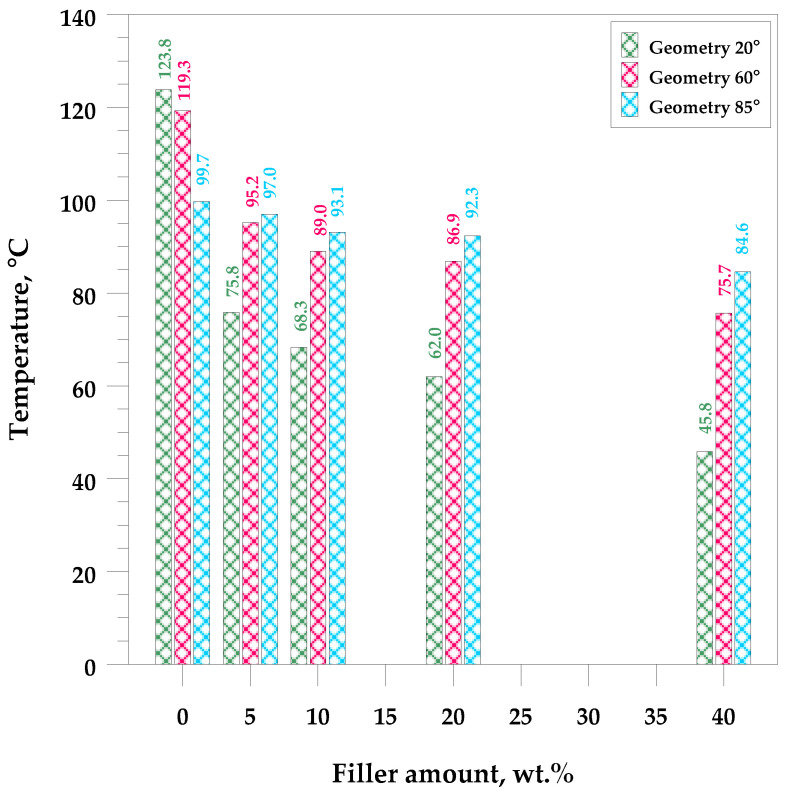
Dependence of the gloss value on the amount of quartz flour in UPR-based composites.

As expected, each filler causes changes in the gloss of the surface [45,46,47]. As was shown in Figure 1, as the content of quartz flour in the hybrid composites increases, the surface of the material becomes more rough and porous, and as a result, the gloss deteriorates (Figure 4). Despite this, it was noticed that even a composite containing 40 wt.% of the filler still has a gloss value above 70 GU for a geometry of 60° and can therefore still be treated as a material with a relatively high gloss.

Changes in the gloss value are not the only differences that characterize the surface of quartz-reinforced composites. Based on the contact angle measurement tests, it was observed that the amount of the filler affects the wettability and also the polarity of the polymer material. 

Increasing the value of the water contact angle ($\theta_{w}$) from 57.0° for pure polyester to 77.9° for the most filled composite (UPR + Q40) causes the obtained hybrid materials to become less susceptible to wetting as the amount of the filler increases and thus become more resistant to water (Table 4). This phenomenon is confirmed by numerous works available in the literature, showing lower liquid sorption in polymer concrete [4].

All the characterized materials showed hydrophilic surface properties due to  θ<90°. Modification of the polyester resin with quartz flour significantly increased the water contact angle, but its value still remained below 90°. The reason for the increase in hydrophobicity is a change not only in the surface topography (roughness) but also in the physicochemical properties of the composites, like polarity.

The situation for measuring the droplet and angle for diiodomethane is opposite to that for water. The value of the contact angle of a CH_2_I_2_ droplet ($\theta_{d}$) slightly but constantly decreased with the amount of quartz flour up to 36.1° for the hybrid composite with 40 wt.% (UPR + Q40). It is noteworthy that this value for pure polyester was 41.7°.

It was noticed that the hybrid composites are characterized by lower surface energy values compared to the pure UPR matrix (Table 4). The SFE decreased with the filler’s incorporation, which can be explained by the presence of a less polar Si–O–Si linkage occurring in the filler [48]. The value drops from 51.17 mJ m^−2^ for pure UPR to 45.63 mJ m^−2^ for the UPR composite with the highest reinforcement of the polyester matrix (UPR + Q40). Reducing the value of the surface free energy causes the material surface to become hydrophobic [48]. A significant change was also noticed for the polar component, the value of which constantly decreased from 12.17 mJ m^−2^ for pure polyester to 5.45 mJ m^−2^ for the composite with the highest quartz packing (UPR + Q40).

## 4. Conclusions

Hybrid composites of different quartz flour amounts and unsaturated polyester resin were obtained and investigated in view of their structure and thermal, mechanical and morphological properties. The results show that the commercially available resin based on the rPET can be used for the preparation of composites with quartz powder up to 40 wt.%. 

Due to the effect of the indirect, improved and more effective heat transfer to the polymer matrix through the dispersed inorganic phase, the reinforced composite materials were characterized by faster decomposition, which ended at slightly lower temperatures.

The hardness and flexural modulus increased with quartz incorporation; meanwhile, the impact strength, flexural strength and strain at break decreased. A small addition of quartz leads to an improvement in the mechanical properties of the polyester composites, while with a high filler content, deterioration results from its agglomeration and the role of particles as stress concentrators.

The gloss values indicate that both the pure resin and its quartz–resin composites belong to the group of high-gloss materials. The surface of the hybrid composites becomes more rough with more pits and holes on it. Due to the presence of a less polar Si–O–Si linkage occurring in the filler, deterioration in the wettability and polarity of the surface was noticed. This made the composite materials more hydrophobic.

This does not change the fact that quartz flour is available and processed with different types of polymer matrices. These composites are a potential alternative material that can be used as artificial stone or marble, monolithic flooring, polymer-modified mortar and concrete or mine charges.

## Figures and Tables

**Table 1:** Surface tensions ($\gamma_{s}$) of test liquids and their dispersive ($\gamma^{d}$) and polar ($\gamma^{p}$) components.

Measuring Liquid	Surface Tension, mJ·m^−2^		
	$\gamma_{s}$	$\gamma^{d}$	$\gamma^{p}$
Diiodomethane	53.2	50.8	2.4
Distilled water	72.8	21.8	51.0

**Table 2:** Thermogravimetric analysis data for the UPR-based composites with quartz flour.

Sample	$T_{5\%}$ ^1^, °C	$T_{10\%}$ ^2^, °C	$T_{50\%}$ ^3^, °C	$T_{max}$ ^4^, °C	Mass Change, %	Residual Mass ^5^, %
pure UPR	317.3	337.9	386.2	382.3; 519.4	−85.04; −13.96	---
UPR + Q5	316.1	337.7	386.0	383.3; 523.4	−83.40; −11.47	4.87
UPR + Q10	316.1	338.1	386.3	383.5; 520.9	−79.21; −11.07	9.24
UPR + Q20	317.5	338.8	388.7	383.8; 514.8	−72.22; −10.05	16.61
UPR + Q40	325.4	345.9	397.2	384.0; 513.8	−62.32; −9.01	28.58

^1^ Temperature of 5% mass loss; ^2^ temperature of 10% mass loss; ^3^ temperature of 50% mass loss; ^4^ maximum decomposition temperature; ^5^ residual mass at 1000 °C.

**Table 3:** Mechanical properties of the quartz–resin hybrid composites.

Sample Name	$a_{cU}$ ^1^, kJ·m^−2^	*HD*^2^, °ShD	$E_{f}$ ^3^, GPa	$\sigma_{f}$ ^4^, MPa	$\varepsilon_{f}$ ^5^, %
pure UPR	8.059	72.1	3.66 ± 0.01	76.58 ± 4.34	2.12 ± 0.11
UPR + Q5	7.892	72.7	3.83 ± 0.01	93.20 ± 7.30	2.51 ± 0.21
UPR + Q10	5.898	73.4	3.97 ± 0.05	79.43 ± 4.03	1.91 ± 0.19
UPR + Q20	4.923	74.6	4.41 ± 0.08	74.40 ± 6.09	1.88 ± 0.08
UPR + Q40	4.133	75.3	5.26 ± 0.04	71.99 ± 0.46	1.41 ± 0.01

^1^ Charpy unnotched impact strength; ^2^ Shore D Hardness; ^3^ flexural modulus; ^4^ flexural strength; ^5^ flexural strain at break.

**Table 4:** Droplet and contact angle measurement value of the measuring liquid.

Sample Name	Droplet and Value of Contact Angle Measurement with Water		Droplet and Value of Contact Angle Measurement with Diiodomethane	
pure UPR	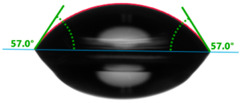	57.0°	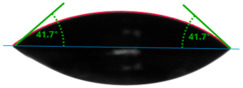	41.7°
UPR + Q5	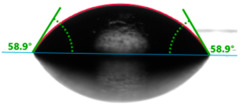	58.9°	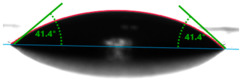	41.4°
UPR + Q10	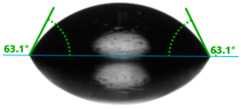	63.1°	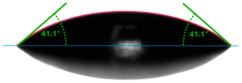	41.1°
UPR + Q20	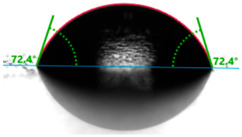	72.4°	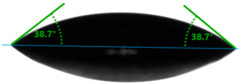	38.7°
UPR + Q40	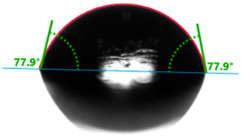	77.9°	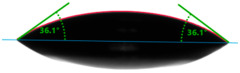	36.1°

## Data Availability

The data presented in this study are available on request from the corresponding author.
